# The Very Virulent IBDV Viral Protein VP3 Promotes the Caspase-3 Mediated Cleavage of GSDME

**DOI:** 10.3390/vetsci13040373

**Published:** 2026-04-13

**Authors:** Tao Zhang, Suyan Wang, Xiaole Qi, Lijie Tang, Yulong Gao

**Affiliations:** 1 State Key Laboratory for Animal Disease Control and Prevention, Harbin Veterinary Research Institute, Chinese Academy of Agricultural Sciences, Harbin 150069, China; zt1079351356@126.com (T.Z.); wangsuyan@caas.cn (S.W.); qixiaole@caas.cn (X.Q.); 2 College of Veterinary Medicine, Northeast Agricultural University, Changjiang Road No. 600, Xiang Fang District, Harbin 150069, China

**Keywords:** IBDV, GSDME, pyroptosis

## Abstract

The infectious bursal disease virus (IBDV) can cause severe immunosuppression and high mortality in chickens, posing a significant threat to the poultry farming industry. Pyroptosis mediated by gasdermin E (GSDME) may be closely related to the tissue damage caused by IBDV. In this study, 3-week-old specific-pathogen-free (SPF) White Leghorns chickens were inoculated intranasally with 1000 copies/200 μL of very virulent IBDV (vvIBDV) Gx strain, and we analyzed GSDME expression in chicken tissues and the cleavage site of GSDME by Caspase-3. Tissue distribution results showed that GSDME and IL-1β transcription in the bursa of Fabricius and kidneys were significantly upregulated by more than five-fold (*p* < 0.01) following vvIBDV infection, indicating a close association with vvIBDV-induced tissue lesions. Further studies demonstrated that Caspase-3 could cleave GSDME at conserved sites (D270), releasing the active N-terminal fragment (GSDME-N) to induce pyroptosis. Furthermore, although IBDV proteins cannot directly cleave GSDME, the viral protein VP3 enhances Caspase-3-mediated GSDME cleavage, thereby triggering pyroptosis. The above results reveal the role of GSDME-dependent pyroptosis in the pathogenesis of IBDV and provide new ideas for the prevention and control strategies against IBDV.

## Simple Summary

GSDME is one of the members of the GSDM family and has been reported as the main pyroptosis protein induced by IBDV for pyroptosis. This study clarifies the expression pattern of GSDME in chicken tissues infected with very virulent IBDV and identifies D270 as the specific cleavage site of chicken Caspase-3 on GSDME, confirming that the N-terminal fragment of GSDME induced by this cleavage is the key to triggering pyroptosis. We further demonstrate that while the IBDV-encoded proteins VP2, VP3, and VP4 can all interact with GSDME, none of these viral proteins directly cleave GSDME; rather, VP3 is the primary viral factor that augments Caspase-3-mediated GSDME cleavage. These results further elucidate the mechanism by which IBDV promotes GSDME cleavage, enrich our understanding of the molecular interactions between IBDV viral proteins and host cell pyroptosis, and provide experimental evidence and new research directions for clarifying the pathogenic process of IBDV and developing targeted prevention and control strategies against IBDV infection.

## 1. Introduction

Infectious bursal disease virus (IBDV) is a significant member of the double-stranded RNA virus family [1]. It mainly infects young chickens and causes an acute, highly contagious disease characterized by necrosis and atrophy of bursa lymphocytes [2,3,4]. It can cause severe immunosuppression and high mortality rates in chicken flocks, making them vulnerable to secondary infections by various pathogens [5,6]. This leads to continuous and significant economic losses for the global poultry farming industry. In terms of the pathogenic mechanism, IBDV mainly targets immune organs, especially replicating extensively in the bursa and spleen, triggering lymphocyte apoptosis and necrosis, accompanied by a significant inflammatory response [7,8,9]. Additionally, the virus can also replicate in tissues such as the kidneys and thymus, causing corresponding tissue pathological damage, such as kidney swelling, urate deposition, and thymus atrophy, further exacerbating the systemic pathological process [10,11]. Based on pathogenicity, IBDV can be divided into classical/attenuated IBDV (cIBDV), variant strain IBDV (varIBDV), and very virulent IBDV (vvIBDV). Compared with classical strains, vvIBDV causes more severe bursal necrosis, systemic tissue damage, intense inflammation, and high mortality, leading to catastrophic economic losses in the poultry industry. Although the pathogenic mechanisms of IBDV have been widely studied, whether vvIBDV-induced severe tissue injury is associated with GSDM-mediated pyroptosis remains largely unclear.

Pyroptosis, a programmed cell death executed by Gasdermin (GSDM) proteins, leads to cell swelling, membrane permeabilization, and the release of inflammatory mediators such as IL-1β and lactate dehydrogenase (LDH) [12,13,14]. Among the Gasdermin family members, GSDMD is cleaved by inflammatory caspases and plays a central role in infections [15,16,17], while GSDME is predominantly cleaved by Caspase-3, converting apoptotic signals into pyroptotic outcomes under conditions such as chemotherapy or certain viral infections [18,19]. Thus, the Caspase-3-GSDME axis represents a key pathway linking apoptosis and pyroptosis. However, although previous studies have shown that IBDV infection induces pyroptosis through GSDME [20], the expression dynamics of GSDME in tissues and the potential involvement of viral proteins in regulating this cleavage remain to be clarified.

Based on this, this study first examined the expression of GSDME in different tissues of SPF chickens after vvIBDV infection. The results showed that the transcriptional level of GSDME was significantly upregulated in the tissues where IBDV replicated significantly. Additionally, the transcriptional levels of IL-1β in the bursa of Fabricius and kidneys were also positively correlated with GSDME. Further, through point mutations and truncated expression, it was determined that Caspase-3 specifically cleaves GSDME at D270 and releases the N-terminal fragment to induce pyroptosis. Finally, we found that different virulence IBDV can promote the cleavage of GSDME by its viral protein VP3 through Caspase-3. This study is expected to provide a new perspective for an in-depth understanding of IBDV’s immune evasion and damage mechanisms, and provide a theoretical basis for the development of intervention strategies targeting the cell death pathway.

## 2. Materials and Methods

### 2.1. Cells and Viruses

Human embryonic kidney HEK-293T cells and the chicken embryo fibroblast line DF-1 were grown in Dulbecco’s modified Eagle’s medium (DMEM; Basal Media, Shanghai, China, Catalog No. L190KJ) containing 10% fetal bovine serum (FBS; Sigma-Aldrich, St. Louis, MO, USA, Catalog No. F8687), penicillin (100 U/mL), and streptomycin (100 µg/mL). HEK-293T cells were incubated at 37 °C in a 5% CO_2_ humidified atmosphere, whereas DF-1 cells were maintained at 38.5 °C under the same CO_2_ conditions. Chicken lymphoma cells (DT40) were cultured in RPMI-1640 medium (Sigma-Aldrich, St. Louis, MO, USA) supplemented with 10% fetal bovine serum (FBS), 2% chicken serum, 1% sodium pyruvate, and 0.1% β-mercaptoethanol. DT40 cells were incubated at 37 °C in a 5% CO_2_ humidified atmosphere. The very virulent Gx strain employed in this work was previously isolated and stored by the Avian Immunosuppressive Disease Research Group at the Harbin Veterinary Research Institute, Chinese Academy of Agricultural Sciences.

### 2.2. Plasmid Construction and Transfection

The genes encoding IBDV structural and non-structural proteins were amplified from the IBDV Gx template. After digestion with *EcoR* I and *Cla* I enzymes, Polymerase Chain Reaction (PCR) amplicons were ligated into pCAGGS to generate the HA-C-VP1, HA-C-VP2, HA-C-VP3, HA-C-VP4, and HA-C-VP5 vectors. Full-length cDNAs of chicken Caspase-3 and GSDME were amplified from the reverse-transcribed cDNA derived from DF-1 cells. The GSDME cDNA was then inserted into a modified pCAGGS-Flag-N-C vector, the Caspase-9 cDNA was inserted into a modified pCAGGS-Flag-C vector, and the Caspase-3 cDNA was inserted into a modified pCAGGS-Myc-C.

HEK-293T cell transfection was conducted using the PolyJet^TM^ (Signagen, Frederick, MD, USA, Catalog No. SL100688) in vitro DNA according to the manufacturer’s instructions.

DF-1 cell transfection was conducted using TransIT-X2 (Mirusbio, Madison, WI, USA, Catalog No. MIR 6000) according to the manufacturer’s instructions.

### 2.3. Antibodies and Reagents

Mouse anti-HA monoclonal antibody (mAb) (Catalog No. H9658), mouse anti-Flag mAb (Catalog No. F1804), mouse anti-Myc mAbs (Catalog Nos. M4439 and C3956), and mouse anti-β-actin mAb (Catalog No. A1978) were purchased from Sigma-Aldrich (St. Louis, MO, USA). All primary antibodies were diluted for Western blot (WB) at 1:2000 and for immunofluorescence assay (IFA) at 1:500, as optimized in our hands.

Secondary antibodies included goat anti-Mouse IgG (H + L) cross-adsorbed, Alexa Fluor 546-conjugated (Catalog No. A-11003) and goat anti-Rabbit IgG (H + L) cross-adsorbed, Alexa Fluor 488-conjugated (Catalog No. A-11008) from Invitrogen (Carlsbad, CA, USA), as well as IRDye 800CW goat anti-Mouse IgG (Catalog No. 926-32210) and IRDye 680RD goat anti-Rabbit IgG (Catalog No. 926-68071) from LI-COR (Lincoln, NE, USA). For WB, IRDye-conjugated secondaries were used at 1:15,000; for IFA, Alexa Fluor-conjugated secondaries were used at 1:500.

### 2.4. Co-Immunoprecipitation (Co-IP) and Western Blotting

For co-immunoprecipitation (Co-IP) assays, HEK-293T cells were co-transfected with the indicated plasmids. At 24 h post-transfection, cells were lysed in 1 mL ice-cold NP-40 lysis buffer (50 mM Tris-HCl, pH 7.4, 150 mM NaCl, 1% NP-40, 1 mM EDTA, and protease inhibitor cocktail) for 30 min on ice. Cell lysates were centrifuged at 12,000× *g* for 15 min at 4 °C, and the supernatant was collected. A total of 1 mg of protein from the supernatant was used per IP reaction. Before immunoprecipitation, the lysate was pre-cleared with 30 μL of protein A/G agarose beads (Abmart, Shanghai, China, Catalog No. A10001) for 1 h at 4 °C to reduce non-specific binding. After pre-clearing, 2 μg of the indicated primary antibody (or isotype-matched mouse IgG as a negative control) was added to the lysate, followed by incubation with gentle rotation for 4 h at 4 °C. Subsequently, 30 μL of protein A/G agarose beads was added, and the mixture was incubated overnight at 4 °C with gentle rotation. Beads were washed four times with ice-cold lysis buffer, and bound proteins were eluted by boiling in 2 × SDS sample buffer for 10 min. Eluted proteins were analyzed by Western blotting. For interaction validation, cells transfected with an empty vector were processed in parallel as an additional negative control to rule out non-specific binding.

For Western blotting analysis, the protein lysates were electrophoretically separated on 12.5% SDS-PAGE gel, and then the proteins were transferred to nitrocellulose membranes. First, the membranes were blocked with 5% (*w*/*v*) skim milk in PBST at 37 °C for 1 h, and then incubated with the corresponding primary antibody diluted with PBS. After washing four times with PBST, an appropriate incubation with an appropriate secondary antibody diluted with PBS was used. Finally, the membrane was washed four times in PBST and imaged using the Odyssey Infrared Imaging System (LICOR BioSciences, Lincoln, NE, USA) for further analysis.

### 2.5. Confocal Microscopy

HEK-293T cells seeded in 35 mm dishes (Biosharp, Hefei, China, Catalog No. BS-20-GJM) were co-transfected with the specified plasmids and incubated for 24 h. Subsequently, the cells were rinsed three times with PBS and fixed using 4% (*v*/*v*) paraformaldehyde (Biosharp, Hefei, China, Catalog No. BL539A) for 30 min. Following fixation, the cells were incubated with the appropriate primary antibodies, followed by incubation with the corresponding fluorescently labeled secondary antibodies. Finally, nuclei were counterstained with DAPI (Vectorlabs, Newark, CA, USA, Catalog No. H-1200) for 10 min, and images were acquired using a Leica SP2 confocal microscope (LSM980, Zeiss, Jena, Germany).

### 2.6. RT-qPCR

Whole-cell RNA was extracted from transfected cells at the indicated time points using the RNAiso Plus kit (Takara Bio Inc., Otsu, Shiga, Japan, Catalog No. 9109). The extracted RNA was reverse transcribed into cDNA using HiScript II QRT SuperMix for quantitative PCR (qPCR) (Vazyme Biotech Co., Nanjing, China, Catalog No. R223-01). The RT-qPCR amplification reaction utilized the SYBR Green qPCR Kit (Toyobo Co., Ltd., Osaka, Japan, Catalog No. QPS-201) with the following cycling host factor conditions: initial denaturation at 95 °C for 2 min, followed by 40 cycles of 95 °C for 5 s and 60 °C for 30 s, followed by a melt curve analysis. The results were analyzed using the 2^−ΔΔCT^ method.

The primer sequences are as follows: *GSDME* (F: 5′-CAAAGGGCTGTGTGGAAAGC-3′, R: 5′-AGCCCCAGAGTACATCCCAT-3′); *β-actin* (F: 5′-CAACACAGTGCTGTCTGGTGGTA-3′, R: 5′-ATCGTACTCCTGCTTGCTGATCC-3′); *IL-1β* (F: 5′-TTTTTGAGCCCGTCACCTTC-3′, R: 5′-AGCACTTCTGGTTGATGTCG-3′).

### 2.7. Ethics Statement

Specific pathogen-free (SPF) White Leghorn chickens were obtained from the Animal Experiment Center of HVRI, Chinese Academy of Agricultural Sciences. The animal experiments were approved by the Animal Ethics Committee of the HVRI (approval no. 240718-03-GR) and conducted in accordance with the international animal welfare standards.

### 2.8. Animal Experiment

Eighteen 3-week-old specific-pathogen-free (SPF) chickens were randomly allocated into two groups with nine birds per group. The experimental group was intranasally inoculated with 1000 viral copies of IBDV strain Gx in a 200 µL volume, while the control group received an equal volume of sterile phosphate-buffered saline (PBS) via the same route. At 3, 4, and 5 days post-infection, SPF chickens were euthanized, and samples were collected aseptically from the bursa of Fabricius, thymus, liver, spleen, and kidney from both groups.

### 2.9. Lactate Dehydrogenase Release Assay

The LDH assay kit (Solarbio, Beijing, China, Catalog No. BC0685) was purchased from Solarbio; the assay reagent was added according to the instructions from the manufacturer, and the optical density (OD) at 450 nm was read in the microplate reader.

### 2.10. Flow Cytometric Analysis of Cell Death Using PI

Digest and collect the cells. Resuspend the collected cells in PBS to achieve a concentration of 1 × 10^6^ cells per milliliter. Add 5 μL of PI solution, incubate at room temperature in the dark for 5 to 10 min, filter through a 40-micron filter, and immediately analyze them on a flow cytometer to detect PI-positive cells.

### 2.11. Statistical Analysis

Statistical analysis was conducted with GraphPad Prism (version 10). Data are expressed as mean ± SD. Differences between groups were assessed using unpaired two-tailed Student’s *t*-tests, one-way ANOVA, or two-way ANOVA, with Dunnett’s post hoc test applied when suitable.

## 3. Results

### 3.1. The Transcriptional Levels of GSDME and IL-1β Were Positively Correlated in the Tissues Where vvIBDV Replicated

**Figure 1:**
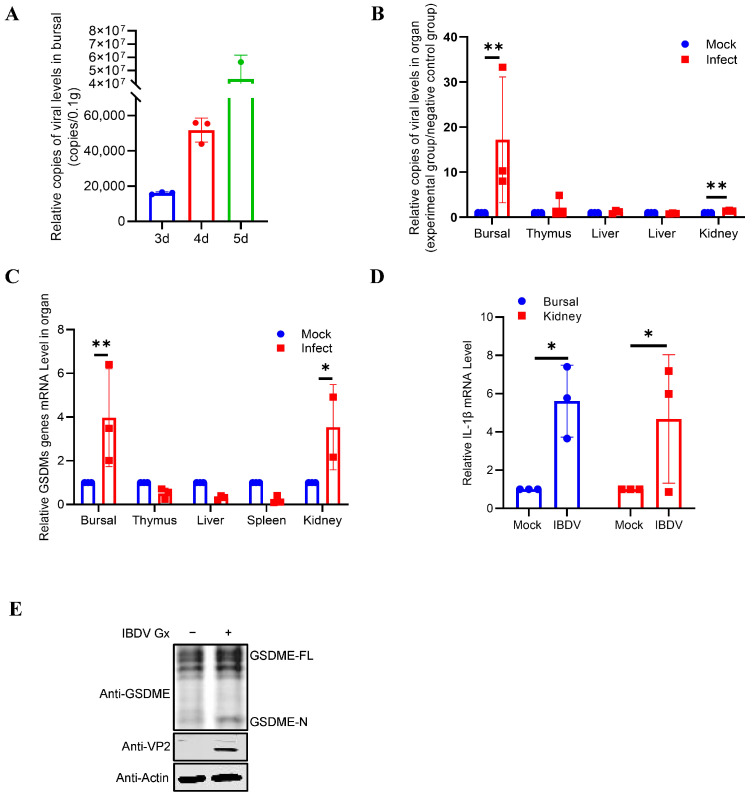
Detection of tissue distribution of GSDME in SPF chickens infected with vvIBDV. (**A**) The viral copy number in the bursa of Fabricius at different time points after IBDV infection (3 days, 4 days, and 5 days). The viral load in the bursa of Fabricius was detected by an absolute fluorescence quantitative method at different infection times, and reached the peak on the 5th day. (**B**) The relative levels of viral copy number in different organs and tissues after infection. (**C**,**D**) The relative expression levels of GSDME mRNA in different immune organs and tissues after infection. The transcription levels of GSDME and IL-1β in each organ on the 5th day after IBDV infection were detected. The expression was significantly upregulated in the bursa of Fabricius and kidney, while there was no significant change in the thymus, liver, and spleen. (**E**) Gx infection induces the activation of GSDME in DT40 cells. Proteolytic cleavage of GSDME in Gx-infected DT40 cells was determined by Western blotting at 24 h post-infection (hpi). The abundance of Gx VP2 protein and β-actin as an internal control was also determined by Western blotting. The figures show the mean ± standard deviation, *n* = 3, * *p* < 0.05, ** *p* < 0.01. (Original image is in Supplementary Materials Figure S1).

To clarify the tissue tropism of vvIBDV infection and its relationship with the expression of molecules related to pyroptosis, we first collected the bursa of Fabricius at 3, 4, and 5 days post-infection (dpi) following Gx (a very virulent IBDV strain) infection and detected the viral load. The RT-qPCR results showed that the viral load reached its peak at 5 dpi (Figure 1A), and then samples of the thymus, liver, spleen, and kidney were collected at 5 dpi for viral load detection. The results of RT-qPCR indicated that the virus replicated significantly in the bursa of Fabricius and the kidneys, while the loads in the thymus, liver, and spleen were relatively low (Figure 1B). To explore whether the expression of GSDME and inflammatory cytokine IL-1β was related to the degree of viral replication, RT-qPCR was further used to detect the transcription levels of these two molecules in the above tissues. The results indicated that in the bursa of Fabricius and kidneys, where viral replication was most active, the transcription levels of GSDME were significantly upregulated and showed a positive correlation with local viral load (Figure 1C). Notably, the mRNA levels of IL-1β were also markedly elevated in the bursa and kidneys (Figure 1D). To further confirm the activation of GSDME in vitro, we infected DT40 cells with Gx for 24 h and then examined the activation of GSDME. The results of Western blotting indicated that GSDME was significantly cleaved in DT40 cells after Gx infection (Figure 1E). Therefore, these results suggest that the replication of vvIBDV in target organs may directly or indirectly activate GSDME and related inflammatory responses.

### 3.2. IBDV Proteins VP2, VP3, and VP4 Interact with GSDME but Cannot Directly Cleave It

**Figure 2:**
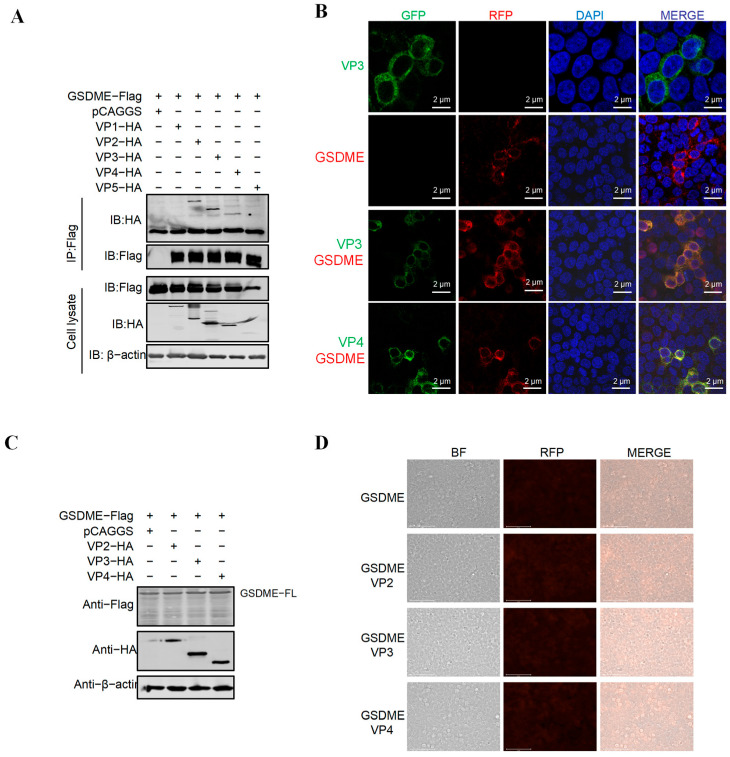
The IBDV viral protein cannot directly cleave GSDME. (**A**) Co-immunoprecipitation (Co-IP) analysis of the interaction between GSDME and IBDV viral proteins (VP1, VP2, VP3, VP4, VP5). HEK-293T cells were co-transfected with Flag-tagged GSDME and HA-tagged IBDV individual viral proteins. Cell lysates were immunoprecipitated with anti-Flag antibody, and the precipitated proteins were detected by Western blotting using anti-HA antibody. The results show that GSDME interacts with VP2, VP3, and VP4, but not with VP1 or VP5. (Original image is in Supplementary Materials Figure S2) (**B**) Confocal assays were used to assess the colocalization between GSDME and VP3 and VP4. HEK-293T cells were co-transfected with pFlag-GSDME and pHA-VP3 or pHA-VP4 for 36 h. Cells were incubated with anti-Flag mAb produced in mice and anti-HA mAb produced in rabbits; the interaction between GSDME and VP3 or VP4 was determined by confocal analysis. (**C**) The IBDV viral protein cannot directly induce pyroptosis through GSDME. HEK-293T cells were co-transfected with pFlag-GSDME and eukaryotic plasmids expressing viral protein-HA fusions containing VP2, VP3, and VP4 of vvIBDV. Twenty-four hours post-transfection, cell lysates were examined by Western blotting using anti-Myc, anti-HA, anti-Flag, and anti-β-actin antibodies; endogenous β-actin expression was used as an internal control. (Original image is in Supplementary Materials Figure S2) (**D**) After transfection of cells with pFlag-GSDME and eukaryotic plasmids expressing viral protein–HA fusions containing VP2, VP3, and VP4 of vvIBDV. PI staining was performed. After separately transfecting each of the three plasmids into HEK-293T cells for 24 h, PI staining was carried out at room temperature for 5 min, and then observed under a fluorescence microscope. The scale bar is 75 μm.

Numerous viruses encoding proteolytic enzymes induce cellular pyroptosis via the direct cleavage of GSDMD or GSDME, including SARS-CoV-2 [16], Seneca Valley virus (SVV) [21], and foot-and-mouth disease virus (FMDV) [22]. VP4, a protein encoded by IBDV, has been identified as a serine protease [23]. To investigate whether IBDV viral proteins can directly interact with GSDME and cleave GSDME to induce pyroptosis, we co-expressed GSDME with individual HA-tagged IBDV proteins (VP1, VP2, VP3, VP4, or VP5) from the Gx strain in HEK-293T cells and assessed their interactions by co-immunoprecipitation (Co-IP). The Co-IP results showed that IBDV viral proteins VP2, VP3, and VP4 interacted with GSDME (Figure 2A). Confocal microscopy further indicated that GSDME co-localized with VP3 and VP4 in the cytoplasm.

To further determine whether IBDV viral proteins can directly cleave GSDME to induce pyroptosis, we co-transfected pFlag-GSDME with pCAGGS-HA plasmids encoding Gx strain VP2, VP3, or VP4 into HEK-293T cells. Western blotting analysis demonstrated that the tested IBDV-encoded proteins lacked the ability to directly cleave GSDME (Figure 2C). As shown in Figure 2D, the PI staining results of the co-transfected cells also showed that there was no specific red fluorescence (the pyroptosis cells showed red fluorescence). Overall, the results indicate that IBDV VP2, VP3, and VP4 interact with GSDME but are unable to directly cleave this protein.

### 3.3. Chicken Caspase-3 Specifically Cleaves GSDME at Position D270, Thereby Inducing Pyroptosis

**Figure 3:**
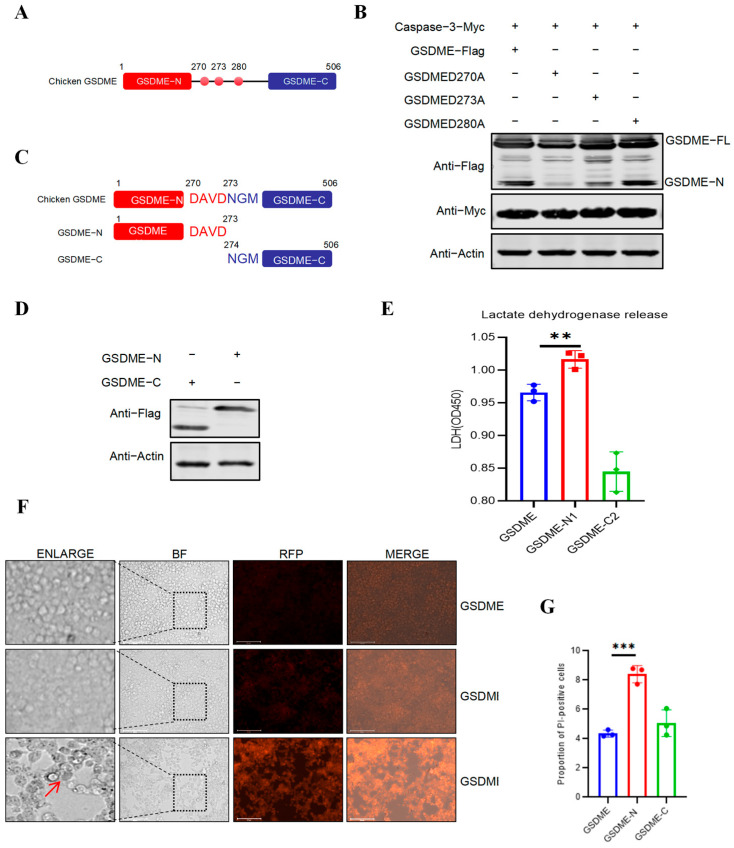
Identification of the specific Caspase-3 cleavage site of GSDME. (**A**) Schematic diagram showing aspartic acid residues in the middle domain of GSDME. (**B**) Detection of Caspase-3 cleavage ability after GSDME aspartic acid site mutation. pGSDME_D270A_, pGSDME_D273A_, and pGSDME_D283A_ and co-transfected them with Caspase-3 into HEK-293T cells to detect the GSDME-N fragment. (Original image is in Supplementary Materials Figure S3) (**C**) Schematic diagram of truncated expression pattern before and after the GSDMED270 mutation site. (**D**) Verify the expression of the N-terminal and C-terminal regions of GSDME. (Original image is in Supplementary Materials Figure S3) (**E**) LDH release from the cells transfected with pMyc-Caspase-3, pFlag-GSDME and pHA-VP3 was measured as described in Materials and Methods. (**F**) After transfection of cells with pGSDME, pGSDME-N, and pGSDME-C, PI staining was performed. HEK-293T cells were transfected with each plasmid separately for 24 h, stained with PI for 5 min at room temperature, and observed under a fluorescence microscope. Red arrows indicate pyroptotic cells. The scale bar is 125 μm. (**G**) The proportion of PI-positive cells was detected using flow cytometry. After collecting the cells, PI was added and stained for five minutes at room temperature. The figures show the mean ± standard deviation, *n* = 3, ** *p* < 0.01, *** *p* < 0.001.

Given that Caspase-3 typically cleaves at the aspartic acid residues within the conserved motif, we identified that the middle domain of chicken GSDME contains three aspartic acid sites (D270, D273, and D283) (Figure 3A). To verify the site at which avian Caspase-3 cleaves GSDME, we generated alanine substitution mutants at each position (pGSDME_D270A_, pGSDME_D273A_, and pGSDME_D283A_), and co-transfected them with Caspase-3 into HEK-293T cells. Western blotting analysis revealed that mutating the aspartic acid residue at position 270 in avian GSDME to alanine (pGSDMED270A) would prevent the cleavage action of Caspase-3 (Figure 3B), while GSDME with mutations at the other two aspartic acid sites could still be cleaved by Caspase-3. These results indicate that, similar to the sites where human and mouse Caspase-3 cleave GSDME, the site where avian Caspase-3 cleaves GSDME is also located at the 270th aspartic acid. Subsequently, to verify that the fragment of GSDME that is cleaved and released by Caspase-3 can induce pyroptosis, based on the identified mutation sites, we constructed truncated expression bodies of the N-terminal and C-terminal fragments of GSDME (Figure 3C), and transfected them into HEK-293T cells to detect their ability to induce LDH release and membrane rupture. The results of Western blotting showed that both the GSDME-N and GSDME-C constructs were successfully expressed (Figure 3D). The results of LDH release showed that GSDME-N could significantly induce the release of lactate dehydrogenase from the cell supernatant (Figure 3E). Similarly, the results of PI staining showed that the cells transfected with GSDME-N exhibited the typical characteristics of pyroptosis (membrane swelling) (Figure 3F). The results of flow cytometry further confirmed that the GSDME-N-transfected cells could significantly increase the proportion of PI-positive cells (Figure 3G). In conclusion, the above results indicate that the specific cleavage site of Caspase cleaving GSDME is the aspartic acid at Asp270.

### 3.4. Viral Protein VP3 of vvIBDV Enhances Caspase-3-Mediated Cleavage of GSDME

**Figure 4:**
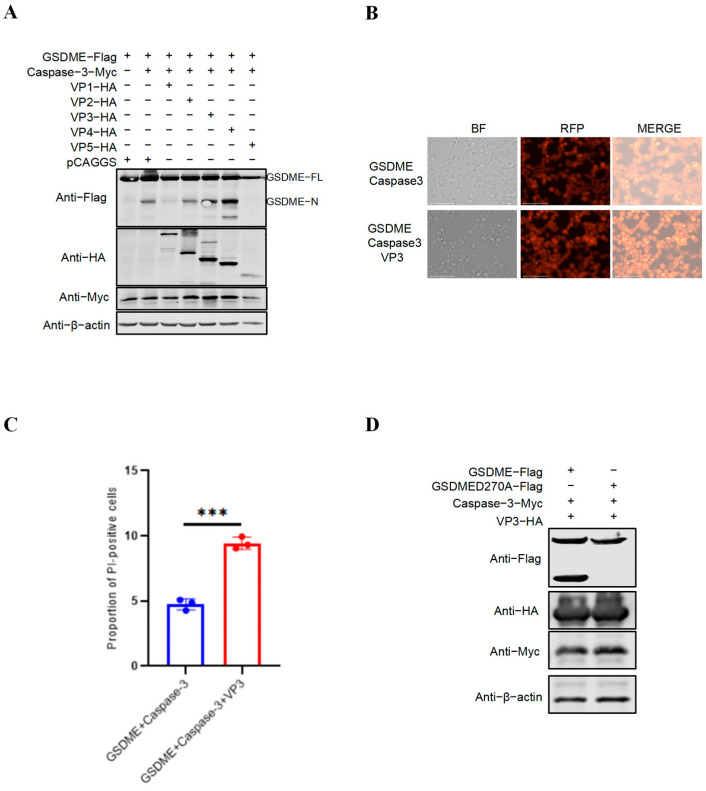
VP3 promotes Caspase-3-mediated cleavage of GSDME. (**A**) Effects of IBDV proteins on Caspase-3-mediated GSDME cleavage were detected by Western blot. HEK-293T cells were co-transfected with pMyc-Caspase-3, pFlag-GSDME, and eukaryotic plasmids expressing viral protein-HA fusions containing VP1, VP2, VP3, VP4, or VP5 of IBDV. Twenty-four hours post-transfection, cell lysates were examined by Western blotting using anti-Myc, anti-HA, anti-Flag, and anti-β-actin antibodies; endogenous β-actin expression was used as an internal control. (Original image is in Supplementary Materials Figure S4) (**B**) PI staining showing pyroptotic cells in cultures expressing Caspase-3, GSDME, and VP3. After transfection of cells with pFlag-GSDME, pMyc-Caspase-3 and pHA-VP3 of vvIBDV. PI staining was performed. After separately transfecting each of the three plasmids into HEK-293T cells for 24 h, PI staining was carried out at room temperature for 5 min, and then observed under a fluorescence microscope. The scale bar is 75 μm. (**C**) Flow cytometry analysis after PI staining. (**D**) Detection of cleavage of GSDME and GSDME_D270A_. HEK-293T cells were co-transfected with pMyc-Caspase-3, pFlag-GSDME or pFlag-GSDME_D270A_ and VP3. Twenty-four hours post-transfection, cell lysates were examined by Western blotting using anti-Myc, anti-HA, anti-Flag, and anti-β-actin antibodies; endogenous β-actin expression was used as an internal control. (Original image is in Supplementary Materials Figure S4) The figures show the mean ± standard deviation, *n* = 3, *** *p* < 0.001.

To verify whether the IBDV viral proteins could promote the Caspase-3 mediated cleavage of GSDME, the pCAGGS-HA eukaryotic expression plasmids of Gx viral proteins (VP1, VP2, VP3, VP4 and VP5, respectively) were co-transfected with the pMyc-Caspase-3 and pFlag-GSDME (with Flag tags fused at both N- and C- terminal) into HEK-293T cells, the cleavage of GSDME and PI staining were observed. As shown in Figure 4A, viral protein VP3 enhanced the cleavage of GSDME of Caspase-3-mediated, while other viral proteins did not. Consistently, PI staining verified that the IBDV viral protein VP3 significantly promoted pyroptosis mediated by the Caspase-3-GSDME pathway (Figure 4B). To further verify the above results, we co-transfected pMyc-Caspase-3, pHA-VP3, and pFlag-GSDME or pFlag-GSDME_D270A_ into HEK-293T cells to detect the N fragment of GSDME. The results of Western blotting indicated that VP3 could not promote the cleavage of GSDMED270A mediated by Caspase-3, but could promote the cleavage of the original GSDME (Figure 4C). These results indicate that the viral protein VP3 of vvIBDV can promote the cleavage of GSDME by Caspase-3.

## 4. Discussion

IBDV infection causes severe immunosuppressive symptoms in chickens. The main reason is the massive death of B lymphocytes in the bursa of Fabricius and the widespread occurrence of inflammatory damage. Although apoptosis played a certain role in this process, whether other programmed cell death pathways were also involved in the pathogenic process of IBDV remains unclear. This study provides novel mechanistic insights that extend previous findings on GSDME-mediated pyroptosis during IBDV infection. First, we identified D270 as the conserved and specific cleavage site of chicken GSDME by Caspase-3, which is critical for generating the active GSDME-N fragment that induces pyroptosis. Second, we revealed that viral protein VP3 acts as a key enhancer to promote Caspase-3-mediated GSDME cleavage, representing a previously unreported viral strategy to regulate host pyroptosis. Quantitatively, GSDME transcription was positively correlated with IL-1β (*p* < 0.01) and upregulated more than five-fold in the bursa and kidneys-target tissues of vvIBDV replication. Notably, this regulatory mechanism is closely associated with the exceptional pathogenicity of very virulent IBDV strains, which cause widespread tissue damage, intense inflammation, and high mortality in global poultry flocks. Our findings therefore not only clarify the molecular basis of vvIBDV-induced immunopathology but also offer a potential target for developing novel intervention strategies against this economically devastating viral disease.

Pyroptosis is essentially inflammatory, characterized by rapid plasma membrane rupture and the release of pro-inflammatory cytokines. The core pathogenic feature of IBDV infection leads to bursal atrophy and a strong inflammatory response, which is similar to the typical characteristics of pyroptosis. Therefore, the molecules involved in the pyroptosis of B lymphocytes in the bursa of Fabricius induced by IBDV may provide new targets for the prevention and treatment of IBDV infection. It is reported that IBDV infection can induce pyroptosis in DF-1 cells mediated by GSDME [20], but the tissue distribution of GSDME after IBDV infection has not yet been clarified. In this study, we characterized the in vivo tissue expression pattern of GSDME in chickens infected with very virulent IBDV, identified D270 as the conserved Caspase-3 cleavage site of chicken GSDME, and demonstrated that viral protein VP3 promotes Caspase-3-mediated GSDME cleavage. These findings further clarify the regulatory mechanism of GSDME-dependent pyroptosis in the pathogenesis of vvIBDV. Human or mouse GSDME is widely expressed in the placenta, heart, brain and kidney [24]. Our data indicate that the gene expression levels of GSDME and IL-1β are positively correlated in the tissues where IBDV replicates. This tissue-dependent and virus-replication-dependent expression pattern suggests that the pyroptosis mediated by GSDME is not an accidental phenomenon, but is directly related to the active replication of the viruses. The increased expression of GSDME in these tissues may reflect the host’s defense response to clear infected cells through inflammatory death, or it may be the result of the virus manipulating the host’s gene expression to promote its own release or exacerbate inflammation. At the same time, as IL-1β is a typical product of the inflammasome and Gasdermin activity, its elevated level confirms the activation of this pathway and indicates that it will exacerbate the inflammatory environment in the tissue, thereby aggravating pathological damage. Although our data have confirmed that the Caspase-3-GSDME axis plays a central role in driving tissue damage, the upstream regulatory mechanisms that trigger this response still require further investigation. Based on previous studies, two potential regulatory pathways can be hypothesized. The NF-κB signaling pathway is a typical main regulatory factor for the pro-inflammatory response during viral infection. The strong induction of IL-1β strongly indicates that NF-κB is activated during IBDV infection [25], which in turn may upregulate pro-inflammatory genes, including GSDME, through transcription. Additionally, viral non-coding RNAs, such as microRNAs (miRNAs), are another possible regulatory layer. Viral miRNAs have been proven to be able to regulate host cell death and immune pathways during infection [26]. It would be meaningful to study whether any vvIBDV-encoded miRNAs will target the GSDME promoter or its regulatory network. Clarifying these upstream triggering factors can not only fill gaps in our current understanding at the mechanistic level but also provide new targets for therapeutic intervention.

In mammals, the activation of GSDME depends on the cleavage of the hinge region that connects the N-terminal and C-terminal [27]. Most viruses encoding proteases can directly and accurately or inaccurately cleave GSDMD/E to induce or inhibit pyroptosis. For example, the 3C protease of foot-and-mouth disease virus (FMDV) [22] and Seneca Valley virus (SVV) [21]. Our results indicate that although IBDV encodes the serine protease VP4, it does not possess the ability to directly cleave GSDME to induce pyroptosis. This indicates that IBDV may mediate pyroptosis by activating the upstream of GSDME. Caspase-3, as a key executor protein for apoptosis and pyroptosis, can be activated through both endogenous and exogenous apoptotic pathways [28,29], and specifically cleaves the 270th aspartic acid of GSDME, releasing its N-terminal active fragment (GSDME-N). This fragment can bind to and disrupt the cell membrane, inducing pyroptosis, resulting in the release of intracellular contents and an inflammatory response [18]. Our results confirm that the site at which chicken Caspase-3 cleaves GSDME is also located at the 270th aspartic acid. The conservation of the D270 cleavage site in the GSDME of birds and mammals highlights its central role in the Caspase-3-GSDME axis. This conserved site was identified in the chicken system and provided an accurate molecular target for viral regulation. Furthermore, we found that the vvIBDV protein VP3 enhanced the cleavage of Caspase-3 on GSDME to induce pyroptosis, indicating that the virus has evolved the ability to utilize this conserved host pathway. It is worth noting that the screening results of this study for the currently predominant novel variant strains of IBDV show that its VP3 could also promote the cleavage of GSDME by Caspase-3. This suggests that this might be the common mechanism of pathogenic IBDV. This mechanism has also been reported in other viruses, such as enterovirus 71 (EV71) [30] and HIV [31]. Thus, it can be seen that the viruses encoding proteases can also activate the cleavage of GSDME by Caspase-3 through other mechanisms, thereby inducing pyroptosis. This highlights the significance of the Caspase-3-GSDME axis, which serves as a conserved host pathway that is often directly or indirectly exploited by various viruses to regulate pyroptosis and host inflammatory responses.

In this study, we demonstrated that the VP3 protein of vvIBDV promotes Caspase-3-mediated cleavage of GSDME, yet the detailed molecular mechanism remains to be further elucidated. First, it will be critical to explore how VP3 enhances the catalytic activity of Caspase-3 toward GSDME, for instance, by direct interaction or by stabilizing the Caspase-3-GSDME complex. Second, the pathological contribution of the Caspase-3-GSDME axis to IBDV-induced tissue injury and immunosuppression needs to be validated in vivo. Finally, the conservation of this regulatory mechanism across different IBDV strains and its potential for targeted intervention also warrant further investigation.

## 5. Conclusions

In conclusion, this study elucidated the pathogenic mechanism of vvIBDV inducing pyroptosis through the Caspase-3-GSDME pathway and identified VP3 as a key viral regulatory factor. This discovery provided a new perspective for understanding the immunosuppression and tissue damage caused by IBDV, and laid a theoretical foundation for developing intervention strategies targeting host cell death pathways.

## Data Availability

The original contributions presented in this study are included in the article/Supplementary Material. Further inquiries can be directed to the corresponding author.

## Supplementary Materials

The following supporting information can be downloaded at: https://www.mdpi.com/article/10.3390/vetsci13040373/s1, Figure S1: Original data for the detection of GSDME activation induced by IBDV. Figure S2: Original data for the interaction between chicken GSDME and IBDV proteins, the detection of the cleavage ability of IBDV VP2, VP3 and VP4 on GSDME. Figure S3: Original data for the detection of the cleavage ability of IBDV VP2, VP3 and VP4 on GSDME. Figure S4: Original data for the GSDME-cleaving activity of Caspase-3 toward wild-type and mutant GSDME, the expression of GSDME N-terminal and C-terminal fragments, the effect of IBDV structural proteins on Caspase-3-mediated GSDME cleavage and the effect of VP3 on Caspase-3-mediated cleavage of wild-type and mutant GSDME.
