# Supplementary material for: The Very Virulent IBDV Viral Protein VP3 Promotes the Caspase-3 Mediated Cleavage of GSDME
Source: Vet Sci. 2026 Apr 13;13(4):373. doi: 10.3390/vetsci13040373 (PMC13120087; doi:10.3390/vetsci13040373)
Supplement: Supplementary file 1 [file vetsci-13-00373-s001.zip › vetsci-4219068-supplementary.pdf]

Supplementary Materials: The Very Virulent IBDV Viral Protein VP3 Promotes the Caspase-3 Mediated Cleavage of GSDME

Tao Zhang [1,2], Suyan Wang [1], Xiaole Qi [1], Lijie Tang [2,*] and Yulong Gao [1,*]

[1] State Key Laboratory for Animal Disease Control and Prevention, Harbin Veterinary Research Institute, Chinese Academy of Agricultural Sciences, Harbin 150069, China; zt1079351356@126.com (T.Z.); wangsuyan@caas.cn (S.W.); qixiaole@caas.cn (X.Q.)
[2] College of Veterinary Medicine, Northeast Agricultural University, Changjiang Road No. 600, Xiang Fang District, Harbin 150069, China
* Correspondence: tanglijie@163.com (L.T.); gaoyulong@caas.cn (Y.G.)

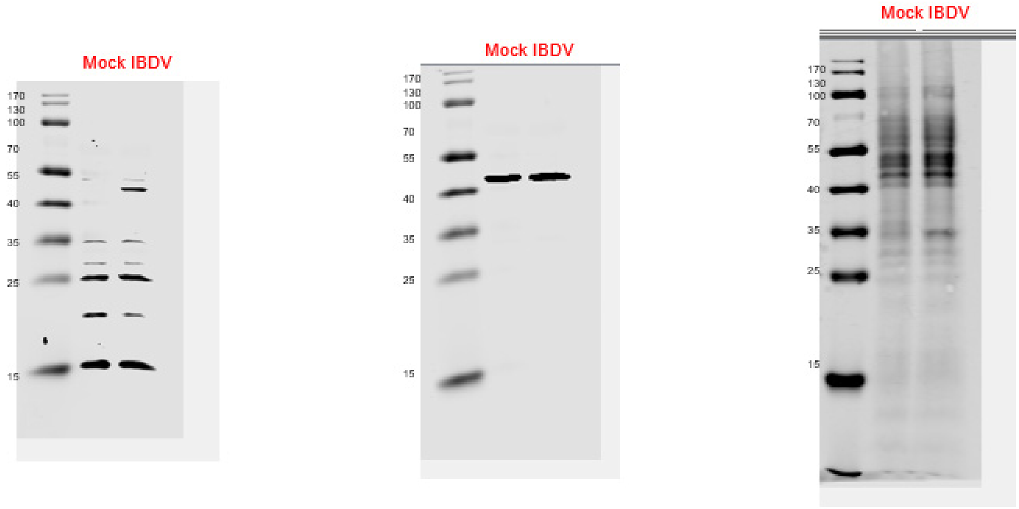

Figure S1: Original data for the detection of GSDME activation induced by IBDV.

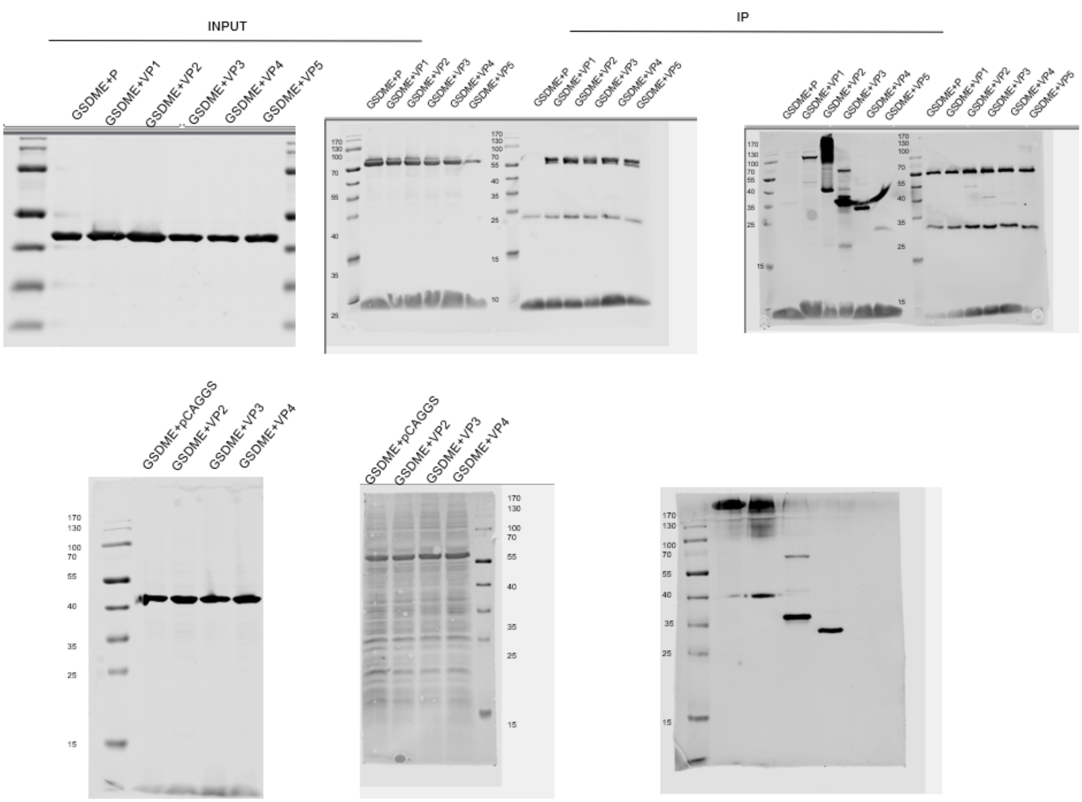

Figure S2: Original data for the interaction between chicken GSDME and IBDV proteins, the detection of the cleavage ability of IBDV VP2, VP3 and VP4 on GSDME.

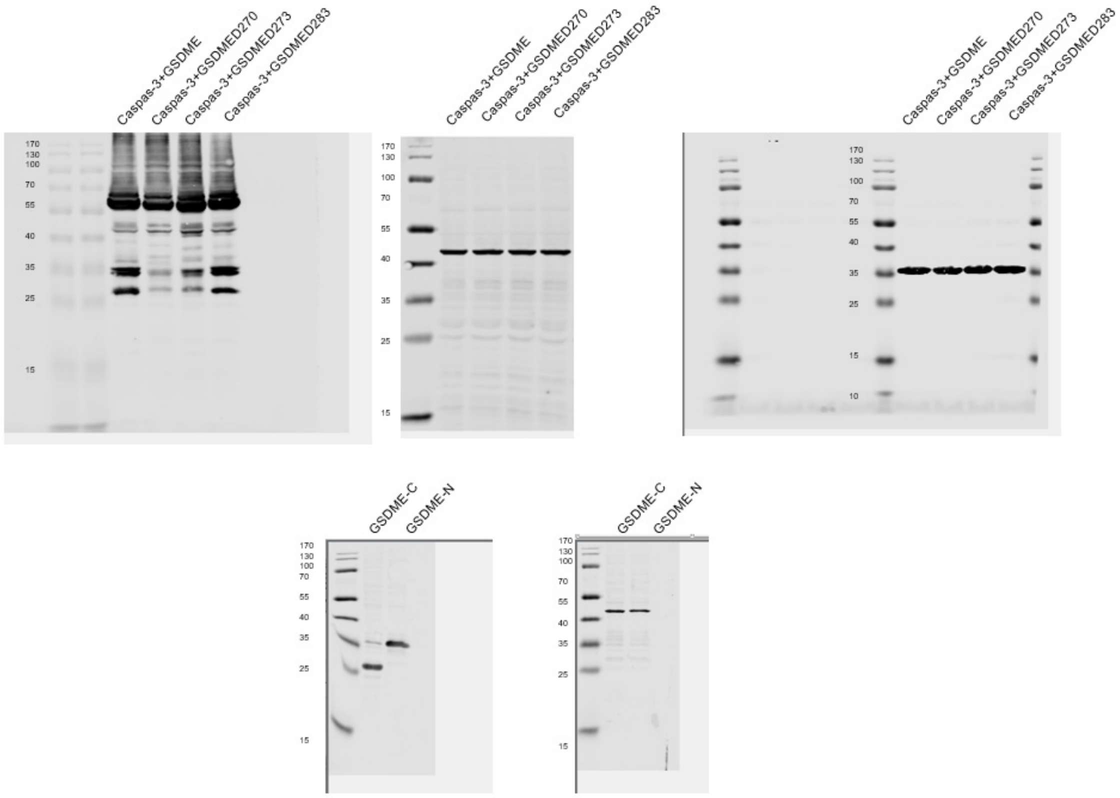

Figure S3: Original data for the detection of the cleavage ability of IBDV VP2, VP3 and VP4 on GSDME.

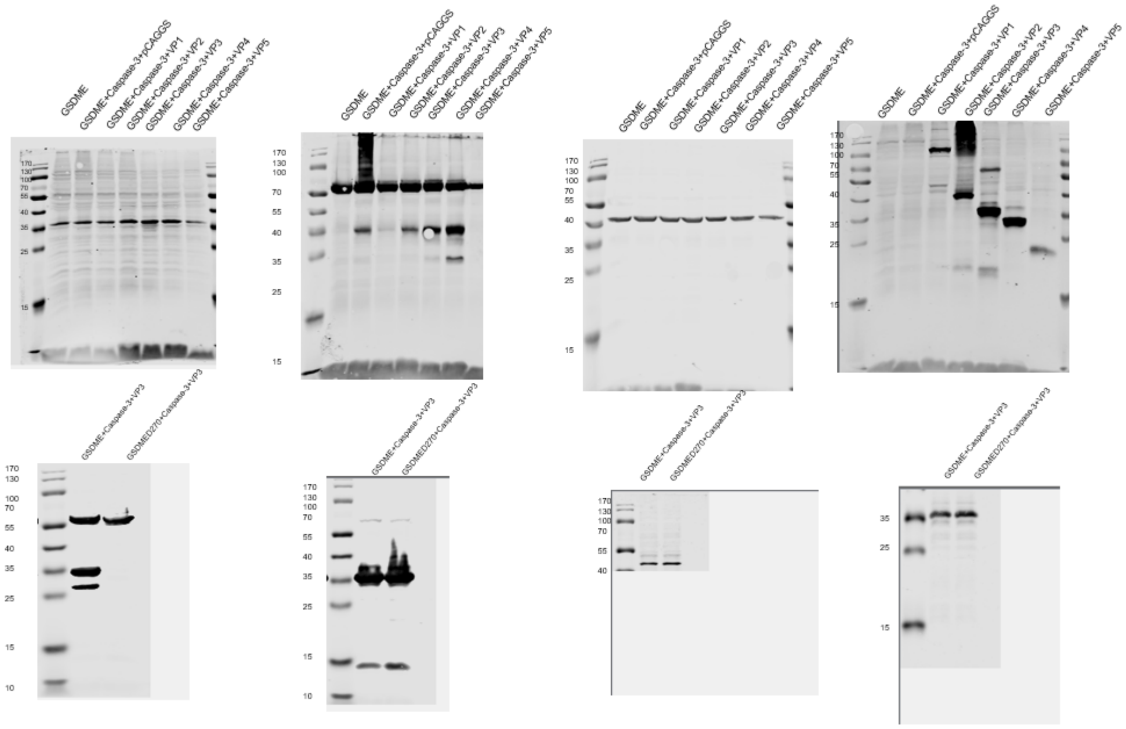

Figure S4: Original data for the GSDME-cleaving activity of Caspase-3 toward wild-type and mutant GSDME, the expression of GSDME N-terminal and C-terminal fragments, the effect of IBDV structural proteins on Caspase-3-mediated GSDME cleavage and the effect of VP3 on Caspase-3-mediated cleavage of wild-type and mutant GSDME.
